# Puberty and disease activity in JIA

**DOI:** 10.1186/1546-0096-12-S1-P151

**Published:** 2014-09-17

**Authors:** Philomine Van Pelt, Anita Hokken-Koelega, Radboud Dolhain, Hans Bijlsma, Aike Kruize, Nico Wulffraat

**Affiliations:** 1 Pediatric Pmmunology and Rheumatology, UMC Utrecht, Utrecht, Netherlands; 2 Rheumatology and Pediatric Pheumatology, Erasmus MC, Rotterdam, Netherlands; 3 Pediatric Endocrinology, Erasmus MC, Rotterdam, Netherlands; 4 Rheumatology, Erasmus MC University, Rotterdam, Netherlands; 5 Rheumatology, UMC Utrecht, Utrecht, Netherlands

## Introduction

Delayed puberty and decreased final length is reported in chronic diseases like Crohn’s disease and JIA with a disease onset at prepubertal age. This may be due to systemic effects of inflammation, undernutrition or medication, for example glucocorticoids or MTX. Treatment with anti TNF has shown to restore delayed growth in JIA.

## Objectives

To describe growth, onset and progression of puberty in established JIA patients who are treated intensively.

## Methods

All consecutive JIA patients aged 10-24 years were asked to participate in this observational follow-up study. Demographic and disease related items were obtained yearly as well as Tanner puberty stages: Pubic Hair Girls (PHG), Breast stage (Bre), Menarche (Men), Pubic Hair Boys (PHB), Genital Stage (Gen). Reference Values were obtained from the Dutch National Growth Study. Median age at reaching each pubertal stage was estimated by Kaplan Meier survival estimates based on the data from patients of Caucasian origin and younger than 21 years. Non parametric tests are used to determine significant differences.

## Results

118 girls (67%) and 58 boys (33%) entered the study. Thirteen percent have systemic onset type of JIA, 24% oligo- persistent type, 54% oligo-extended and polyarticular type and 9% other subtypes of JIA.

Median disease duration is 8.5 years (IQR 7,3). Median JADAS 27 is 3,8 (IQR 6.9), active joint count is 0,0 (2.0), DAS 28 is 2,18 (1,37). MTX is ever or currently used in 78% of the patients, anti TNF in 15% and systemic corticosteroids in 24%. Early disease onset before the age of 8 years is present in 59% of the patients. Eleven patients are of non-Caucasian origin and 19 patients are older than 21 years, and are excluded from growth and puberty analysis. Median SDS length is -0,29 (IQR 1,38), SDS weight -0,27 (1,46), SDS BMI -0,08 (1.71). PHG, Bre, PHB and Gen are delayed in all stages 2-5, more pronounced in stage 5. Median delay in PHG stage 5 is 3,4 years, Bre stage 5 3,4 years, Menarche 3,5 years, PHB stage 5 1.6 years and Gen stage 5 1.7 years. Progression of puberty is more delayed in stage 4 and 5 compared to healthy Dutch children. No significant differences are seen between users and non-users of systemic corticosteroids, MTX or anti TNF. Subtype of JIA, disease activity and age at onset of JIA did not significantly influence results.

## Conclusion

Although disease activity is low due to intensive treatment, puberty is still remarkably delayed. Further investigation in clinical relevance and cause of delayed puberty is needed.

## Disclosure of interest

None declared

